# Pan‐Adrenergic Inhibition Alleviates Post‐Hemorrhagic Hydrocephalus by Attenuating Neutrophil Extracellular Traps‐Mediated Glymphatic Dysfunction

**DOI:** 10.1002/cns.71259

**Published:** 2026-11-07

**Authors:** Yijian Yang, Yanjie Zhou, Qian Ouyang, Hanati Nuerlanbieke, Yexin Yuan, Junqiang Wang, Kaiyue Wang, Peng Long, Yifeng Chen, Zhikun Liu, Jun Huang, Zhiping Zhang, Zhangjie Su, Xia Wang, Gelei Xiao

**Affiliations:** ^1^ Department of Neurosurgery, Xiangya Hospital Central South University Changsha Hunan China; ^2^ Diagnosis and Treatment Center for Hydrocephalus, Xiangya Hospital Central South University Changsha Hunan China; ^3^ Hunan International Scientific and Technological Cooperation Base of Brain Tumor Research, Xiangya Hospital Central South University Changsha Hunan China; ^4^ National Clinical Research Center for Geriatric Disorders, Xiangya Hospital Central South University Changsha Hunan China; ^5^ Department of Pediatrics, Xiangya Hospital Central South University Changsha Hunan China; ^6^ Department of Neurosurgery, Zhuzhou Hospital Affiliated to Xiangya School of Medicine Central South University Zhuzhou Hunan China; ^7^ Department of Neurosurgery, Xinjiang Key Laboratory of Neurological Disorder Research The Second Affiliated Hospital of Xinjiang Medical University Urumqi Xinjiang China; ^8^ Department of Neurosurgery, John Radcliffe Hospital Oxford University Hospitals NHS Foundation Trust Oxford UK

**Keywords:** adrenergic storm, aquaporin‐4, glymphatic system, hydrocephalus, intraventricular hemorrhage, neutrophil extracellular traps, PI3K/AKT/mTOR pathway

## Abstract

**Aims:**

Post‐hemorrhagic hydrocephalus (PHH) is a devastating complication. This study aims to elucidate how the acute post‐hemorrhagic adrenergic storm drives neutrophil extracellular traps (NETs) formation, blocks the glymphatic system (GS), and causes an imbalance in intracranial fluid homeostasis, leading to PHH. It is necessary to explore whether pan‐adrenergic inhibition as non‐surgical intervention strategies can rescue this neuro‐immune dysregulation.

**Methods:**

Single‐cell RNA sequencing (scRNA‐seq) was used to draw the immune infiltration map. Utilizing a rat PHH model, ventricular volumes and glymphatic fluid dynamics were assessed via MRI and fluorescent tracer tracking. Multiple behavioral analyses were used to observe the neurological dysfunction. The structural integrity of the GS was assessed using IF staining and TEM. The formation of NETs was detected using ELISA, WB, and IF. In vitro, flow cytometry and molecular assays were used to explore the changes in molecular pathways in epinephrine‐stimulated primary neutrophils, and NETs were used to treat astrocytes to study the interaction between them. A pharmacological intervention using pan‐adrenergic inhibitor cocktail PPA (prazosin, propranolol, atipamezole), NETs agonist, NETs inhibitor, and AKT activator was conducted to explore the mechanism.

**Results:**

scRNA‐seq identified a pathogenic neutrophil subset presented as a transcriptional phenotype consistent with NETs, which dominated hemorrhagic stroke. Structurally, adrenergic storm‐driven NETs extrusion occluded perivascular spaces and stripped aquaporin‐4 (AQP4) polarization at astrocytic endfeet, precipitating glymphatic stasis and severe ventricular dilation. Molecular analysis revealed that epinephrine might promote the formation of NETs by activating the PI3K/AKT/mTOR cascade. PPA blocked this pathological axis, inhibited NETs, polarized AQP4 distribution, restored the fluid dynamics of the GS, and relieved cognitive deficits after intraventricular hemorrhage and alleviated hydrocephalus.

**Conclusion:**

After PHH, targeted pan‐adrenergic inhibition can block the adrenergic storm and inhibit NET formation, protect the clearance function and structural integrity of the GS, stabilize the intracranial fluid homeostasis, and alleviate hydrocephalus in the acute phase. The molecular mechanism may involve the PI3K/AKT/mTOR cascade. This mechanism provides a potential therapeutic strategy for neuro‐immune‐based drug protection.

## Graphical abstract

Intraventricular hemorrhage induces an adrenergic response that activates PI3K/AKT/mTOR signaling and promotes neutrophil extracellular trap (NET) formation, impairing glymphatic clearance and aggravating post‐hemorrhagic hydrocephalus (PHH). Pan‐adrenergic inhibition after hemorrhage reduced PI3K/AKT/mTOR activation and NET formation, preserved AQP4 polarization and glymphatic structural integrity, stabilized intracranial fluid homeostasis, and attenuated acute hydrocephalus. These findings link adrenergic signaling to neutrophil‐driven glymphatic dysfunction and identify it as a therapeutic target.

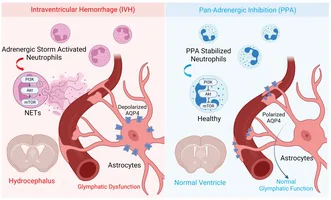

## 1 Introduction

Hemorrhagic stroke (HS), encompassing intracerebral hemorrhage (ICH) and intraventricular hemorrhage (IVH), represents a group of devastating cerebrovascular events characterized by high morbidity, mortality, and profound long‐term neurological deficits [1]. Secondary hydrocephalus is one of the most severe complications following IVH, manifesting as the pathological accumulation of cerebrospinal fluid (CSF) and progressive ventricular dilation [2]. Historically, the pathogenesis of post‐hemorrhagic hydrocephalus (PHH) was predominantly explained by the physical obstruction of the macroscopic CSF pathways by blood clots or hypersecretion of CSF by the choroid plexus [3]. However, this view has been increasingly challenged by evidence showing that PHH is not merely a mechanical consequence of clot obstruction. Instead, a paradigm shift is underway toward a “microvascular, neuroimmune, and glymphatic” perspective, in which erythrocyte lysis, iron toxicity, microcirculatory dysfunction, and robust neuroinflammation converge to impair CSF homeostasis [4, 5].

A critical yet historically underappreciated component of this new paradigm is the “adrenergic storm”—a massive, systemic release of epinephrine and norepinephrine triggered by severe neurotrauma and hemorrhage [6]. Although the adrenergic storm is well recognized in trauma and systemic critical illness, its specific role in IVH/ICH and subsequent hydrocephalus has been largely overlooked. This gap likely stems from the traditional focus of PHH research on macroscopic clot obstruction and choroid plexus hypersecretion, which diverted attention from the neuroendocrine–immune axis. This excessive noradrenergic output deeply impacts central nervous system (CNS) immunity and fluid homeostasis [7]. Concurrently, rupture of the blood–brain barrier (BBB) allows peripheral neutrophils to rapidly infiltrate the brain parenchyma and perivascular spaces (PVS) [8]. Upon exposure to the adrenergic storm and extreme oxidative stress, these infiltrating neutrophils form neutrophil extracellular traps (NETs) [9, 10, 11]. These NETs—composed of decondensed chromatin, histones, and cytotoxic enzymes such as myeloperoxidase (MPO) and neutrophil elastase (NE)—not only exacerbate localized tissue damage but also trap erythrocytes and promote microvascular thrombosis [12, 13]. We therefore propose that the adrenergic–NETs–AQP4 axis may serve as a unifying mechanism linking neuroinflammation, microvascular injury, and glymphatic failure in PHH. This study aims to fill the gap in our understanding of how the adrenergic storm contributes to PHH by systematically testing this hypothesis.

The accumulation of NETs directly assaults the brain's specialized fluid clearance networks, specifically the glymphatic system [14]. The glymphatic system facilitates the convective exchange of CSF and interstitial fluid (ISF), a process heavily dependent on the highly polarized expression of aquaporin‐4 (AQP4) water channels at astrocytic endfeet [15, 16, 17]. We postulate that the adrenergic storm acts as a primary catalyst for massive NET formation, which occludes these delicate perivascular channels, while their associated cytotoxic enzymes chemically degrade the gliovascular interface, leading to severe AQP4 depolarization. Consequently, the synergistic failure of fluid clearance creates a catastrophic fluid stagnation that clinically manifests as secondary hydrocephalus.

The intracellular execution of NET formation under adrenergic stress is increasingly linked to the phosphoinositide 3‐kinase (PI3K)/AKT/mammalian target of rapamycin (mTOR) signaling axis [18]. Although traditionally a neuroprotective survival pathway, hyperactivation of the PI3K/AKT/mTOR cascade in epinephrine‐stimulated neutrophils may serve as a fundamental metabolic prerequisite for chromatin decondensation and active cell death [19, 20].

The primary objective of this study is to systematically delineate the causal mechanisms linking the adrenergic storm, NETs possibly mediated by PI3K/AKT/mTOR, glymphatic system dysfunction, and secondary hydrocephalus. We aim to validate that silencing the adrenergic storm fundamentally dismantles NETs, repolarizes AQP4, and restores macroscopic fluid homeostasis in PHH.

## 2 Methods

### 2.1 Experiment Design and Groups

The rats were randomly divided into sham group (sham operation without autologous blood injection.), sham+PPA (prazosin, propranolol, atipamezole) group (treatment with PPA post sham operation), IVH group (IVH), IVH + PPA group (treatment with PPA post IVH), IVH + PPA + PMA group (NETs activator PMA injection after treatment with PPA post IVH), IVH + DNase I group (treatment with NETs inhibitor DNase I post IVH).

### 2.2 Animals and Ethics

The study included a total of 348 male Sprague Dawley rats weighing between 200 and 250 g (10.5% mortality and 315 rats survived and sacrificed for experiment finally). Details of the numbers of animals used in the experiment are provided in Table S1 These rats were obtained from Xiangya hospital of Central South University. A reverse 12‐h light/12‐h dark cycle environment was used to acclimate the rats, who were also provided with unlimited access to water and food. All procedures followed the guidelines of the Experimental Animal Ethics Committee, Xiangya hospital of Central South University (202509140). This study followed the Animal Research: Reporting of in Vivo Experiments guidelines for reporting animal experiments.

### 2.3 Intraventricular Hemorrhage Model

We established IVH rats' model by injecting autologous blood in the right lateral ventricle as previously described [21]. Detailed establishment process can be seen in Supplementary Methods.

### 2.4 Drug Injection

Pan‐adrenergic inhibitor cocktail contained Prazosin, Propranolol, and Atipamezole (PPA) [7]. The aim was to broadly inhibit adrenergic receptors rather than to target catecholamine release. Prazosin (CM00858, Proteintech, China), Propranolol (CM16191, Proteintech, China), Atipamezole (CM14700, Proteintech, China), PMA (Phorbol 12‐myristate 13‐acetate, HY‐18739, MCE, United States), DNase I (HY‐108882, MCE, United States). Detailed methodological details can be seen in Supplementary Methods. The details of the administration time and the experimental time can be found in Figure S1.

### 2.5 Single‐Cell RNA Sequencing (scRNA‐Seq) Data Analysis

Publicly available scRNA‐seq data was obtained from the GEO database (GSE167593) [22, 23]. Detailed establishment process can be seen in Supplementary Methods.

### 2.6 Magnetic Resonance Imaging

The MRI scanning was performed using a 3.0‐T scanner (Siemens, Germany). T2 sequence (slice thickness 1.2 mm; TR 2780 ms; TE 121 ms) was set during this experiment. T1 sequence (slice thickness 1.2 mm; TR 500 ms; TE 9.3 ms) was set during this experiment immediately after injection of Gadopentetate Dimeglumine (Gd‐DTPA) (H10950231, Consun, China) in cisternal magna at a speed of 5 ul/min for 10 min. The scanning outcomes were observed by MicroDicom DICOM Viewer (MicroDicom Ltd., Bulgaria) and the ventricular systems were subsequently rendered using 3D Slicer (NIH, America) in order to calculate the ventricle volume.

### 2.7 Brain Section, Immunofluorescence (IF) Staining and Transmission Electron Microscopy (TEM)

The primary antibodies used for IF were listed in Table S2. Details are described in Supplementary Methods.

### 2.8 Behavioral Test

We used open field test (OFT), novel object recognition (NOR) and Morris water maze (MWM) to evaluate the neurological function of rats. Details are described in Supplementary Methods.

### 2.9 Intracisternal Magna Injection and in Flow

The brain was removed and sectioned into four coronal brain sections at bregma +1.6 mm, −2.4 mm, −3.2 mm, and −4.0 mm with a thickness of 100 μm using a cryostat microtome [24]. Detailed steps can be seen in Supplementary Methods.

### 2.10 Intrahippocampal Injection and Out Flow

The brain was removed and sectioned into four coronal brain sections at bregma −1.6 mm, −2.4 mm, −3.2 mm, and −4.0 mm with a thickness of 100 μm using a cryostat microtome [24]. Detailed steps can be seen in Supplementary Methods.

### 2.11 Cell Culture, CCK8 and Apoptosis

Details are described in Supplementary Methods.

### 2.12 Elisa

Epinephrine (R‐M‐R19062, RUISAIQI BIOTECHNOLOGY, China) and MPO‐ DNA (R‐M‐R87452, RUISAIQI BIOTECHNOLOGY, China) were detected using the kit according to the instructions.

### 2.13 Western Blot (WB)

The primary antibodies used for WB can be seen in Table S3. A density analysis of the bands was performed using Image J.

### 2.14 Image Analysis

Details are described in Supplementary Methods.

### 2.15 Statistical Analysis

All data are presented as the mean ± SD, and differences between mean values were assessed by one‐way analysis of variance (ANOVA) or two‐way ANOVA, and Tukey's multiple comparison test using GraphPad Prism (GraphPad Software, America). *p* values < 0.05 were considered significant.

## 3 Results

### 3.1 Single‐Cell Sequence Reveals Neutrophils Recruitment and Transcriptional Phenotype Consistent With NET Following Hemorrhagic Stroke

To assess cellular heterogeneity following HS, scRNA‐seq was performed on cells isolated from the brain microenvironment (Figure 1A). UMAP visualization identified major CNS and immune cell populations (Figure 1B,C and Figure S2A,B). Quantitative analysis showed a higher proportion of neutrophils in the HS group compared to control (Figure 1D and Figure S2C–E); however, due to the lack of biological replicate information in the merged dataset, no donor‐level statistical test was performed, and these results are presented descriptively. Unsupervised sub‐clustering of the neutrophil compartment revealed five distinct states (clusters 0–4) (Figure 1E–G and Figure S2F–H). Cluster 0 was observed at a higher proportion in the HS group and exhibited the highest transcriptional NETosis potential score, consistent with a NET‐related activation phenotype (Figure 1H,I).

**FIGURE 1:**
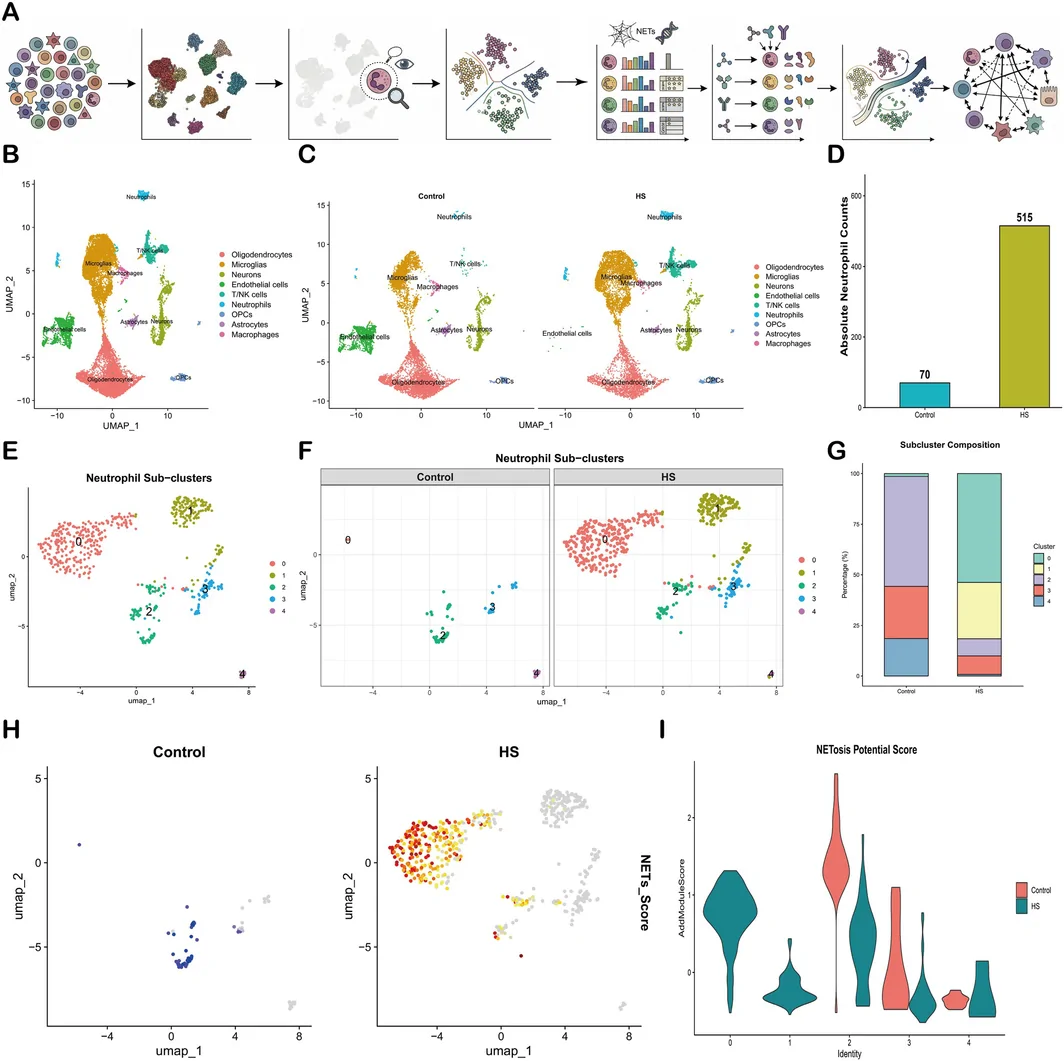
Single‐cell Sequence Reveals Neutrophils Recruitment and Transcriptional Phenotype Consistent with NET Following Hemorrhagic Stroke. (A) Schematic overview of the scRNA‐seq data analysis. (B) UMAP visualization of all integrated cells, colored by major cell types. (C) UMAP visualizations split by group. (D) Absolute counts of neutrophils between two groups. (E) UMAP displaying the re‐clustering of isolated neutrophils into five distinct sub‐clusters (labeled 0 through 4). (F) UMAP plots of the neutrophil sub‐clusters separated by group. (G) Relative percentage composition of the five neutrophil sub‐clusters between two groups. (H) UMAP feature plots displaying the distribution of the NETs Score in Control and HS groups. (I) The NETosis Potential Score across the five neutrophil sub‐clusters (0–4). Data are assessed by one‐way ANOVA or two‐way ANOVA, and Tukey's multiple comparison test is used. HS, Hemorrhagic stroke; NETs, Neutrophil extracellular traps; UMAP, Uniform manifold approximation and projection.

Core marker analysis demonstrated that cluster 0 neutrophils highly expressed activation genes, including Osgin1 and Clec4n (Figure S3A–D). Pseudotime trajectory analysis suggested a transition of neutrophils from early states (Cluster 2) toward cluster 0 (Figure S3E). CellChat analysis predicted that neutrophils act as a signaling hub within the microenvironment (Figures S3F, S4A). These cells were predicted to communicate with microglia, endothelial cells, and astrocytes via specific ligand‐receptor axes, such as Spp1‐Cd44 and Tnf‐Tnfrsf1b, which have been implicated in neuroinflammation in previous studies (Figure S4B–E).

### 3.2 PPA Alleviates Post‐Hemorrhagic Hydrocephalus and Neuroinflammation

Plasma and CSF were collected from rats in the sham and IVH groups to measure temporal changes of epinephrine, thereby characterizing the intracerebral adrenergic storm after IVH (Figure 2A,B and S7A). The results showed that the intracerebral adrenergic storm peaked at 1 day after IVH and then gradually declined. To evaluate the effects of PPA itself on normal rats, we measured blood pressure and heart rate over time in four groups of rats (Figure 2C,D). We found that although PPA exerted blood pressure and heart rate lowering effects, these effects did not reach statistical significance. T2‐weighted MRI and 3D reconstruction were utilized to quantify lateral ventricular volume (Figure 2E). PPA administration in the sham group did not exert any appreciable effects. IVH induced significant ventriculomegaly, which was attenuated by PPA treatment or DNase I treatment, and subsequently exacerbated by PMA administration (Figure 2F,G and Figure S5). Concurrently, immunofluorescence for GFAP and Iba1 indicated pronounced astrocyte and microglia activation in the IVH group (Figure 2H–K). Inhibition of NETs via PPA or DNase I reduced glial reactivity, suggesting that adrenergic‐driven NETs exacerbate secondary hydrocephalus and neuroinflammation.

**FIGURE 2:**
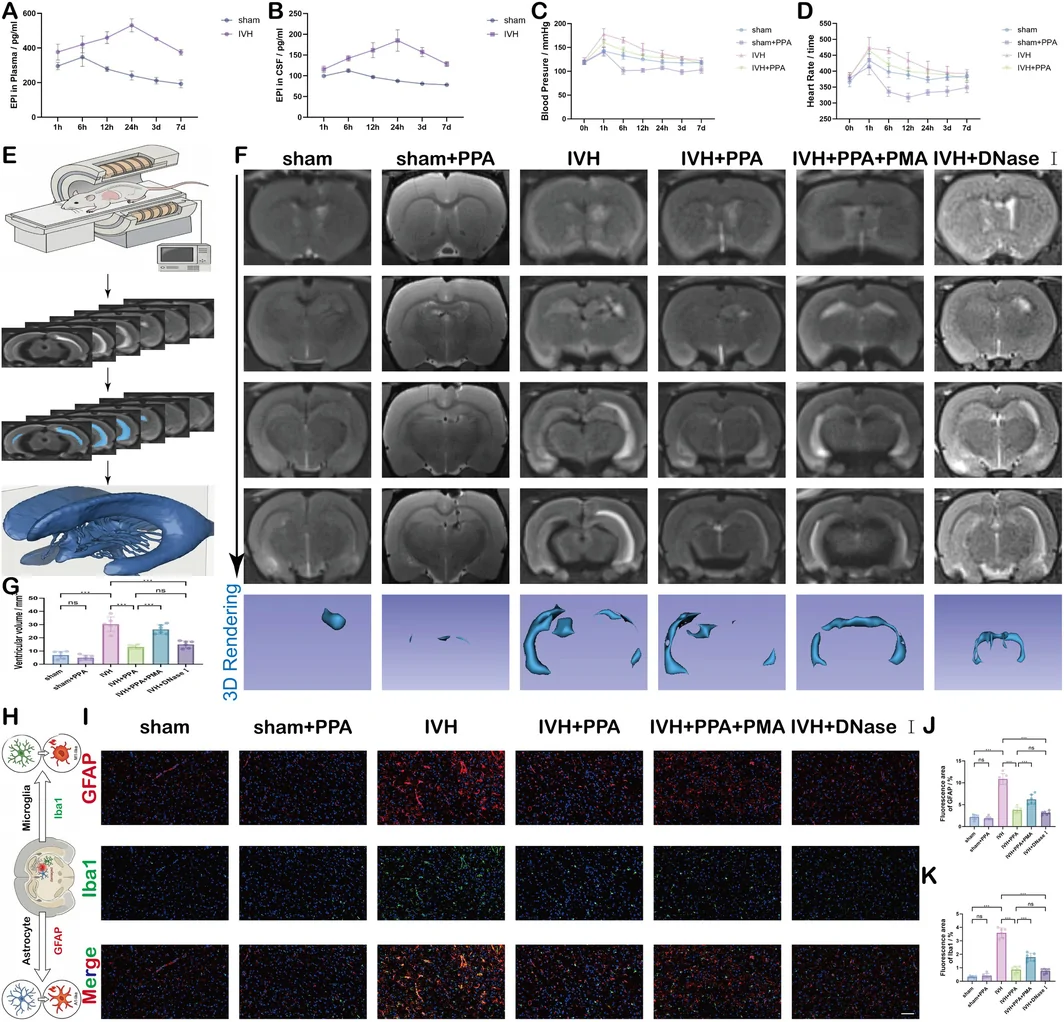
PPA Alleviates Post‐Hemorrhagic Hydrocephalus and Neuroinflammation. (A) Epinephrine in plasma during timelines in sham and IVH. (B) Epinephrine in CSF during timelines in sham and IVH. (C) Blood pressure during timelines in sham, sham+PPA, IVH, IVH + PPA. (D) Heart rate during timelines in sham, sham+PPA, IVH, IVH + PPA. E: Schematic diagram of MRI T2. (F) Representative coronal brain plane and 3D rendering of each group on MRI T2. (G) Ventricular volume measured by rendering of each group. (H) Schematic diagram of brain section. (I) Representative IF for GFAP and Iba‐1 of each group (scale bar = 100 μM). (J) Quantification for GFAP positive area of each group. (K) Quantification for Iba‐1 positive area of each group. All data are presented as mean ± SD (*n* = 6 per group). Data are assessed by one‐way ANOVA or two‐way ANOVA, and Tukey's multiple comparison test is used. Significance levels are denoted as: ***p < 0.001.

### 3.3 Pan‐Adrenergic Inhibition Ameliorates Neurocognitive and Motor Deficits Driven by Post‐Hemorrhagic Hydrocephalus

Neurocognitive and motor outcomes were evaluated using the OFT, NOR, and MWM (Figures 3A, 3C, 3E). PPA did not exert any effect on neurological function in sham group rats. IVH mice exhibited reduced inner zone exploration, decreased object discrimination indices, and impaired spatial navigation compared to sham controls (Figures 3B, 3D, 3F–L). Pan‐adrenergic inhibition with PPA or NETs inhibition with DNase I improved performance across all behavioral metrics, while PMA negated these benefits.

**FIGURE 3:**
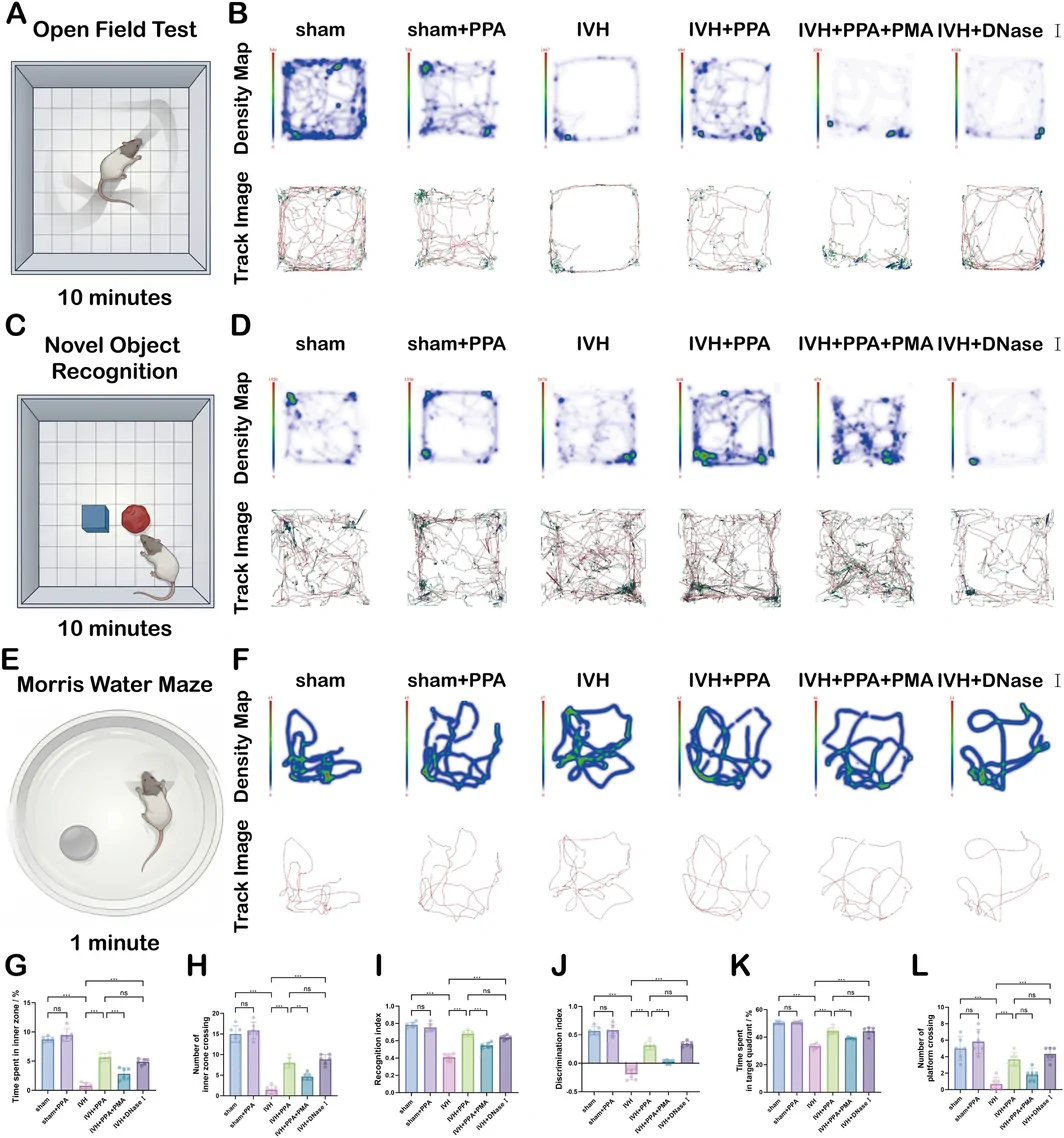
Pan‐Adrenergic Inhibition Ameliorates Neurocognitive and Motor Deficits Driven by Post‐Hemorrhagic Hydrocephalus. (A) Schematic diagram of OFT. (B) Representative density map and track image of each group in OFT. (C) Schematic diagram of NOR. (D) Representative density map and track image of each group in NOR. (E) Schematic diagram of MWM. (F) Representative density map and track image of each group in MWM. (G) Time spent in inner zone of each group in OFT. (H) Number of inner zone crossings of each group in OFT. (I) Recognition index of each group in NOR. (J) Discrimination index of each group in NOR. (K) Time spent in target quadrant of each group in MWM. (L) Number of platform crossings of each group in MWM. All data are presented as mean ± SD (*n* = 6 per group). Data are assessed by one‐way ANOVA or two‐way ANOVA, and Tukey's multiple comparison test is used. Significance levels are denoted as: **p < 0.01, ***p < 0.001.

### 3.4 PPA Restores Glymphatic Cerebrospinal Fluid Influx and Efflux

Glymphatic function was assessed using cisterna magna and intracerebral injections of the fluorescent tracer RITC‐Dextran 70 K. In flow and out flow analyses demonstrated a significant reduction in tracer penetration and clearance in the IVH brain compared to sham (Figure 4A–F). PPA treatment did not induce significant enhancement of glymphatic function in sham group rats. Rats treated with PPA or DNase I exhibited restoration of glymphatic function. MRI using Gd‐DTPA confirmed a severe decline in tracer‐distribution abnormalities. PPA or DNase I administration partially restored glymphatic transport, whereas PMA re‐induced fluid stasis, indicating that NETs deposition obstructs glymphatic conduits (Figure 4G,H).

**FIGURE 4:**
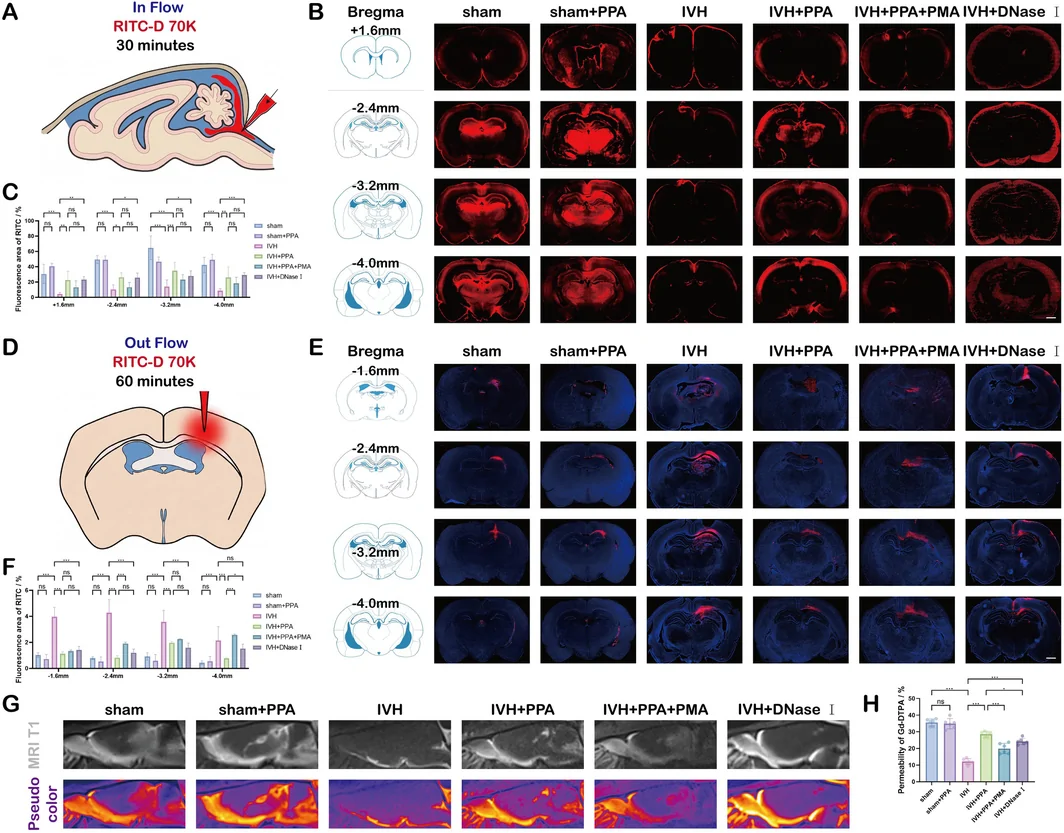
PPA Restores Glymphatic Cerebrospinal Fluid Influx and Efflux. (A) Schematic diagram of In Flow. (B) Representative images for RITC‐D 70 K accumulation in four coronal brain sections at bregma +1.6 mm, −2.4 mm, −3.2 mm, and −4.0 mm of each group (scale bar = 1000 μm). (C) Quantification for fluorescence area covered by RITC‐D 70 K in four sections per animal of each group. (D) Schematic diagram of Out Flow. (E) Representative images for RITC‐D 70 K accumulation in four coronal brain sections at bregma −1.6 mm, −2.4 mm, −3.2 mm, and −4.0 mm of each group (scale bar = 1000 μm). (F) Quantification for fluorescence area covered by RITC‐D 70 K in four sections per animal of each group. (G) Representative sagittal brain plane after injection of Gd‐DTPA processed by pseudo‐color of each group on MRI T1. (H) Quantification for permeability of Gd‐DTPA of each group. All data are presented as mean ± SD (*n* = 6 per group). Data are assessed by one‐way ANOVA or two‐way ANOVA, and Tukey's multiple comparison test is used. Significance levels are denoted as: *p < 0.05, **p < 0.01, ***p < 0.001. Gd‐DTPA, Gadopentetate Dimeglumine; RITC‐D 70 K, Rhodamine B isothiocyanate‐Dextran‐average mol wt‐70,000.

### 3.5 Pan‐Adrenergic Inhibition Rebuilds AQP4 Polarization and Perivascular Space Integrity

Dual‐immunofluorescence showed that IVH leads to a loss of perivascular AQP4 polarization, disrupting its co‐localization with GFAP and CD31 (Figure 5A–D). TEM revealed swollen astrocytic endfeet and physical detachment from the endothelial basement membrane in the IVH group, marked dilatation of the perivascular spaces was observed, accompanied by swelling of astrocytic endfeet, membrane rupture, and physical detachment from the basement membrane (Figure 5E). PPA when administered to normal rats, neither promoted AQP4 polarization nor maintained the integrity of perivascular spaces. PPA or DNase I treatment preserved AQP4 polarity and ultrastructural integrity, demonstrating that NETs mediate the structural degradation of the gliovascular interface necessary for normal CSF transport.

**FIGURE 5:**
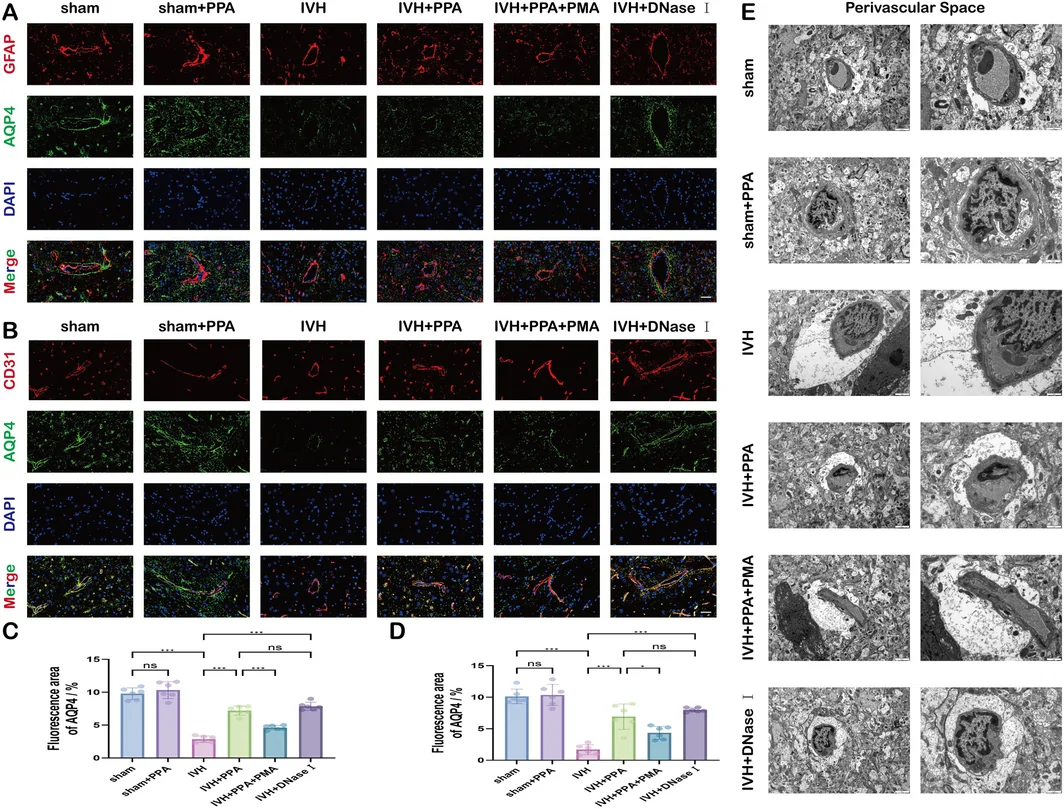
Pan‐Adrenergic Inhibition Rebuilds AQP4 Polarization and Perivascular Space Integrity. (A) Representative IF for GFAP and AQP4 of each group (scale bar = 50 μm). (B) Representative IF for CD31 and AQP4 of each group (scale bar = 50 μm). (C) AQP4 fluorescence intensity around GFAP of each group. (D) AQP4 fluorescence intensity along CD31 of each group. (E) Representative images for TEM in 2500× (scale bar = 2 μm) and 5000× (scale bar = 1 μm) of each group. All data are presented as mean ± SD (*n* = 6 per group). Data are assessed by one‐way ANOVA or two‐way ANOVA, and Tukey's multiple comparison test is used. Significance levels are denoted as: *p < 0.05, ***p < 0.001.

### 3.6 Pan‐Adrenergic Inhibition Suppresses NET Formation

Immunofluorescence staining was used to evaluate the presence of NETs in the brain parenchyma. The co‐localization of Cit‐H3 with MPO and NE was markedly increased following IVH. Treatment with the pan‐adrenergic inhibitor PPA or NETs inhibitor DNase I significantly reduced the fluorescence areas of these NETs markers (Figure 6A–E). ELISA‐based quantification of MPO‐DNA complexes also revealed the same pattern (Figure 6F and Figure S7B). Western blot analysis of Cit‐H3, MPO, and NE protein levels corroborated the histological findings, demonstrating that adrenergic blockade or NETs inhibition attenuates NETs in vivo, an effect that was reversed by the administration of the NETs inducer PMA (Figure 6G–K).

**FIGURE 6:**
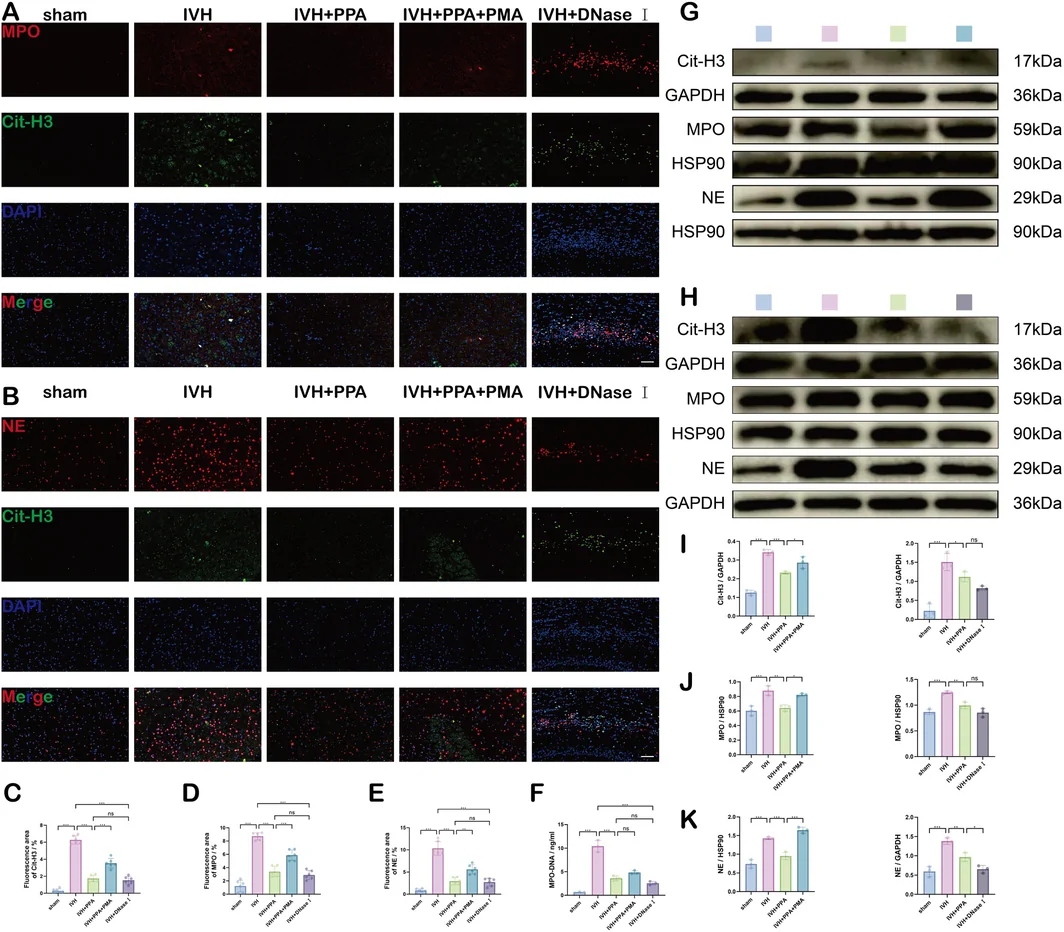
Pan‐Adrenergic Inhibition Suppresses NET formation. (A) Representative IF for MPO and Cit‐H3 of each group (scale bar = 50 μm). (B) Representative IF for NE and Cit‐H3 of each group (scale bar = 50 μm). (C) Quantification for Cit‐H3 positive area of each group. (D) Quantification for MPO positive area of each group. (E) Quantification for NE positive area of each group. (F) MPO‐DNA of each group. (G) Western blot scans for Cit‐H3 and GAPDH, MPO and HSP90, NE and HSP90 of each group in animal experiment 1. (H) Western blot scans for Cit‐H3 and GAPDH, MPO and HSP90, NE and GAPDH of each group in animal experiment 2. (I) Quantification for Cit‐H3 and GAPDH of each group. (J) Quantification for MPO and HSP90 of each group. (K) Quantification for NE and HSP90 or GAPDH of each group. All data are presented as mean ± SD (*n* = 6 per group in IF, and *n* = 3 per group in WB). Data are assessed by one‐way ANOVA or two‐way ANOVA, and Tukey's multiple comparison test is used. Significance levels are denoted as: *p < 0.05, **p < 0.01, ***p < 0.001. Cit‐H3, Histone H3 (citrulline R2 + R8 + R17); MPO, Myeloperoxidase; NE, Neutrophil Elastase.

### 3.7 PPA Suppresses NET Formation by Inhibiting PI3K/AKT/mTOR and NETs Damage the Microstructure of the Glymphatic System

In primary neutrophils (PMNs), cell viability following treatment with different concentrations of epinephrine was assessed using CCK‐8, and NET formation was quantified by MPO‐DNA complexes (Figure 7A,B and Figure S7B). Based on these results, 10 μM was selected for cellular intervention, and Day 3 was selected for detection based on the temporal changes of MPO‐DNA (Figure 7C and Figure S7B). To determine whether the individual interventions exerted similar effects, single‐agent groups were established, and MPO‐DNA quantification and Annexin V/PI staining were performed (Figure 7D–F and Figures S6, S7B). No statistically significant difference was observed between the single agents and the combination treatment in terms of NET formation; however, a difference was observed in overall Annexin V/PI positivity, suggesting that the combination treatment may exert broader effects on cell damage. Flow cytometry demonstrated that epinephrine (Epi) stimulation significantly increased Annexin V/PI positivity, which was mitigated by PPA or DNase I but re‐activated by the AKT agonist SC79 (Figure 7G,H and Figure S6). Western blot analysis showed that Epi upregulated NET markers (Cit‐H3, MPO, NE); PPA or DNase I reduced NET formation, whereas SC79 restored it (Figure 7I,J). MPO‐DNA exhibited the same pattern (Figure 7K and Figure S7B). Alongside increased NET formation, phosphorylation of PI3K/AKT/mTOR was detected (Figure 7L,N). PPA suppressed this kinase activation, while SC79 restored it. Together, these data indicate that adrenergic stimulation induces NET formation as a phenotypic readout, accompanied by increased cell damage measured by Annexin V/PI positivity. The observation that DNase I, which degrades extracellular NETs, reduced Annexin V/PI positivity suggests that NETs may contribute to the observed cell damage. Whether PI3K/AKT/mTOR activation is necessary for this process requires further validation using genetic loss‐of‐function approaches. To further investigate the effects of NETs on the glymphatic system, we stimulated PMNs with PMA, isolated NETs from the supernatant, and used them to treat astrocytes, thereby exploring these effects at the microstructural level (Figure 7R). Following NET treatment, GFAP expression was upregulated, whereas AQP4, a key microstructural component of the glymphatic system, was decreased, along with its membrane‐anchoring protein β‐DG, indicating that NETs disrupt the microstructural integrity of the glymphatic system (Figure 7M,O). NET treatment likewise induced astrocyte apoptosis (Figure 7P,Q and Figure S6). In contrast, DNase I exerted a protective effect on the microstructural integrity of the glymphatic system by degrading extracellular NETs.

**FIGURE 7:**
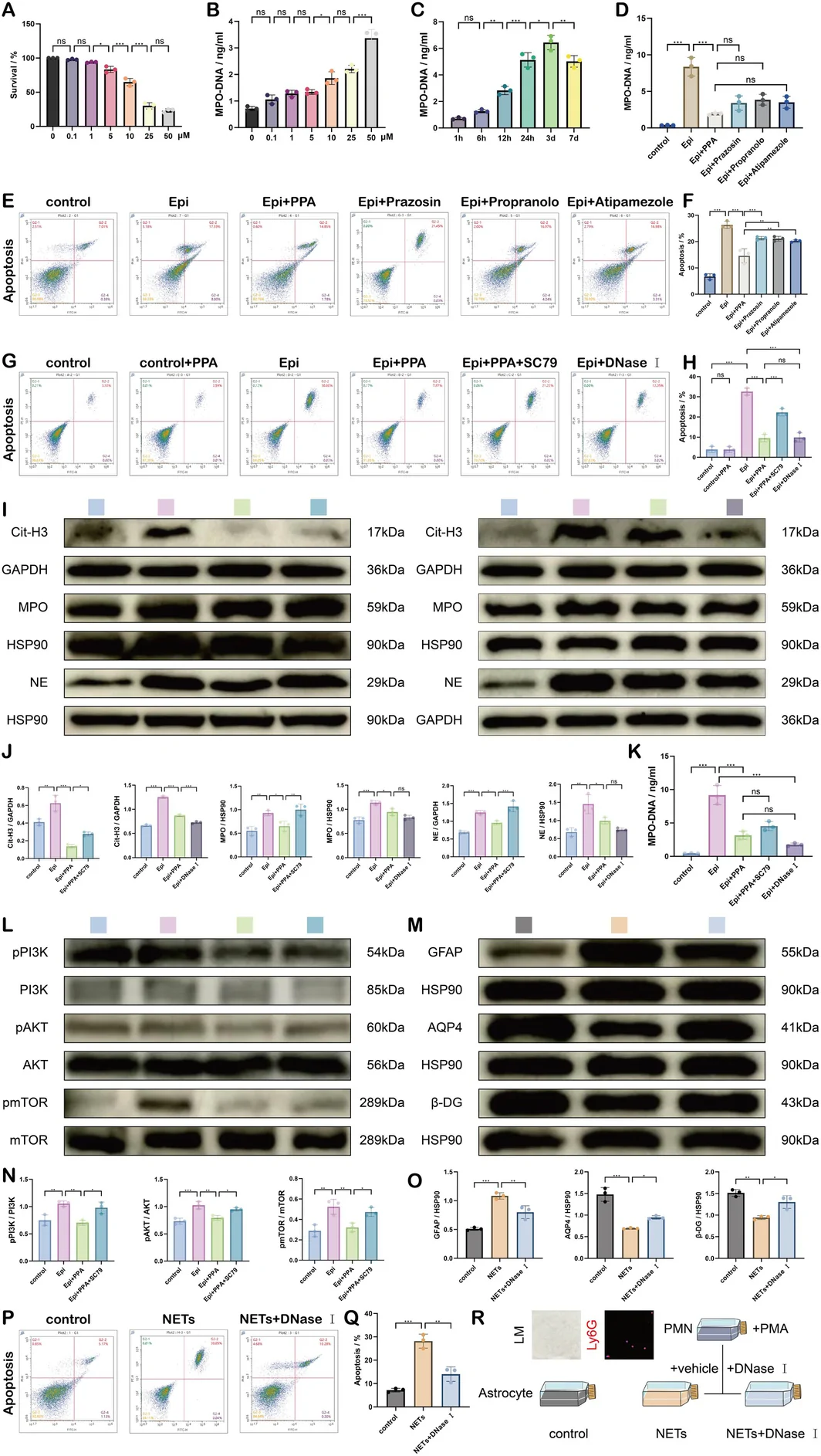
PPA Suppresses NET formation by Inhibiting PI3K/AKT/mTOR and NETs damage the microstructure of the glymphatic system. (A) Survival rate of PMN in different concentrations. (B) MPO‐DNA of PMN in different concentrations. (C) MPO‐DNA of PMN during timelines. (D) MPO‐DNA of PMN in the experiment of individual adrenergic antagonists. (E, F) Representative images for apoptosis in flow cytometry of each group in the experiment of individual adrenergic antagonists. (G, H) Representative images for apoptosis in flow cytometry of each group in cells. (I) Western blot scans for Cit‐H3 and GAPDH, MPO and HSP90, NE and HSP90 or GAPDH of each group in cells. (J) Quantification for Cit‐H3 and GAPDH, MPO and HSP90, NE and HSP90 or GAPDH of each group. (K) MPO‐DNA of PMN in cells. (L) Western blot scans for pPI3K and PI3K, pAKT and AKT, pmTOR and mTOR of each group in cells. (M) Western blot scans for GFAP and HSP90, AQP4 and HSP90, β‐DG and HSP90 of each group in the experiment of astrocytes. (N) Quantification for pPI3K and PI3K, pAKT and AKT, pmTOR and mTOR of each group in cells. (O) Quantification for GFAP and HSP90, AQP4 and HSP90, β‐DG and HSP90 of each group in the experiment of astrocytes. (P, Q) Representative images for apoptosis in flow cytometry of each group in the experiment of astrocytes. (R) Schematic diagram of the experiment of astrocytes. All data are presented as mean ± SD (*n* = 3 per group). Data are assessed by one‐way ANOVA or two‐way ANOVA, and Tukey's multiple comparison test is used. Significance levels are denoted as: *p < 0.05, **p < 0.01, ***p < 0.001. mTOR, Mammalian target of Rapamycin; pAKT, Phospho AKT; PI3K, Phosphatidylinositol 3‐kinase; pmTOR, Phospho mammalian target of Rapamycin; pPI3K, Phospho phosphatidylinositol 3‐kinase.

## 4 Discussion

The current study establishes a comprehensive neuro‐immune axis detailing how the acute post‐hemorrhagic adrenergic storm drives secondary hydrocephalus. IVH and ICH are notorious for initiating a rapid, multifaceted cascade of secondary injuries, driven by erythrocyte lysis, iron toxicity, and severe neuroinflammation. Although physical obstruction of macroscopic CSF pathways by blood clots has historically been considered the primary driver of PHH, our findings pivot the focus toward the microscopic and molecular disruption of the CNS fluid clearance networks. Our macroscopic findings of fluid stasis complement the seminal work by Hussain et al. and Sun et al., who recently demonstrated that acute post‐traumatic edema is fundamentally driven by a systemic noradrenergic surge that suppresses glymphatic clearance [6, 7]. By extending this framework to hemorrhagic stroke, we provide a crucial cellular mechanism by which NETs translate this systemic adrenergic stress into a perivascular blockade.

scRNA‐seq reveals that brain‐infiltrating neutrophils are not a monolithic population but display profound phenotypic plasticity [25]. In alignment with recent transcriptomic profiling of neuroinflammation, we resolved a highly mobilized, pro‐inflammatory neutrophil subcluster dominating the hemorrhagic brain. These findings are highly consistent with emerging literature demonstrating that early mature and activated neutrophils shift toward an aggressive, terminally differentiated state primed for NETs release during cerebral ischemia and traumatic brain injury [26, 27]. In line with recent single‐cell transcriptomic atlases of ischemic stroke, we confirmed the existence of a highly pathogenic, terminally differentiated neutrophil state in the injured brain [28]. Furthermore, extensive profiling has similarly identified that mature neutrophils can undergo rapid phenotypic transitions toward aggressive states during severe stress [29]. Although baseline neutrophils may exhibit reparative or homeostatic features, the adrenergic‐rich microenvironment actively re‐educates these infiltrates toward a destructive, NET‐prone trajectory. Thus, targeting this specific systemic stress response offers more precise therapeutic avenues than broad immune depletion, which carries substantial risks of systemic infection [30].

The selection of Day 3 as the primary endpoint was based on two considerations. First, our experimental data showed that the adrenergic storm peaked at Day 1 after hemorrhage; considering the time required for downstream events such as NET formation and AQP4 depolarization to develop, Day 3 was chosen to capture the maximal impact of this cascade. Second, previous studies on intracranial hemorrhage have commonly used Day 3 as a representative time point for the acute phase, making it a rational and comparable endpoint. This time point also coincides with the early phase of ventricular dilation and glymphatic dysfunction, allowing us to assess the causal relationship between NET formation and CSF dynamics impairment before irreversible structural damage occurs. The physical extrusion of NETs creates a mechanical blockade that fundamentally disrupts CSF dynamics [31]. Our multimodal imaging data, utilizing both MRI and fluorescent tracing, demonstrate that the expansive, mesh‐like deposition of extracellular DNA and histones directly paralyzes glymphatic influx and efflux. This mechanism extends beyond the PVS; NETs heavily interact with surrounding tissues, trapping toxic metabolites and inflammatory cytokines within the brain parenchyma. This concept is elegantly supported by recent breakthrough studies, particularly the work of Zhang et al., who comprehensively demonstrated that NETs mediate the impairment of meningeal lymphatic drainage by directly injuring lymphatic endothelial cells, exacerbating secondary hydrocephalus [14]. Furthermore, Gao et al. demonstrated that NETs mediated by microvesicles promote thrombosis and brain injury [32]. Our research seamlessly integrates with and expands upon their paradigm by identifying the acute adrenergic storm as the apex upstream trigger for this devastating NETosis. Consequently, the enzymatic and physical degradation of these drainage pathways by adrenergic‐driven NETs is a primary catalyst for secondary hydrocephalus.

At the gliovascular interface, the dense accumulation of NETs directly precipitates the loss of AQP4 polarization on astrocytic endfeet. The functional patency of the glymphatic system is critically dependent on this highly polarized AQP4 expression, which facilitates the rapid convective transport of water and solutes across the BBB [33]. Our ultrastructural and immunofluorescence analyses confirm that NETs shear and detach these endfeet, resulting in severe AQP4 depolarization. Enlarged perivascular spaces would compromise the percolation and circulation of intracranial fluid through this microstructural compartment. This microanatomical devastation aligns with broad pathological hallmarks observed across various CNS disorders, as described by Rasmussen et al., where AQP4 polarity loss tightly correlates with the accumulation of neurotoxic proteins and worsened clinical outcomes [34]. In the context of HS, this disruption immediately translates into interstitial edema and ventriculomegaly. Restoring AQP4 polarity therefore remains a critical bottleneck in reinstating convective glymphatic clearance and relieving interstitial edema.

Recent advances have identified both meningeal lymphatic damage and choroid plexus hypersecretion as important contributors to PHH pathogenesis [35, 36]. The question naturally arises: how do these pathological events relate to our observed NET‐mediated perivascular obstruction? We propose a hierarchical model in which NETs occupy an upstream position in the injury cascade. Zhang et al. elegantly demonstrated that NETs directly injure meningeal lymphatic endothelial cells and promote lymphatic thrombosis, leading to impaired drainage and exacerbated secondary hydrocephalus after IVH. Their work identified CX3CR1 as a key mediator of NET‐induced lymphatic endothelial damage. Our findings extend this framework by identifying the adrenergic storm as the apex trigger upstream of NET formation—a mechanism that Zhang et al. did not address. In our model, the adrenergic surge drives massive NET extrusion, which then produces two spatially and temporally distinct consequences [37]: (1) perivascular blockade of glymphatic channels in the brain parenchyma, leading to local fluid stagnation and AQP4 depolarization (our findings), and (2) downstream injury to meningeal lymphatic vessels, impairing CSF outflow at the dural level (Zhang et al.). Regarding choroid plexus hypersecretion, this mechanism may represent a compensatory response to impaired CSF clearance rather than an independent initiating event [38]. When glymphatic and meningeal lymphatic drainage are blocked by NETs, the choroid plexus may upregulate CSF production as a compensatory attempt to maintain intracranial homeostasis—a maladaptive response that paradoxically exacerbates ventricular dilation. Under this hierarchical model, NETs function as the “common upstream node” that simultaneously disables multiple fluid clearance pathways, and targeting the adrenergic–NETs axis could therefore alleviate PHH more effectively than approaches that address only one downstream component [39].

On an intracellular level, the execution of NETs and subsequent parenchymal apoptosis might be driven by the hyperactivation of the PI3K/AKT/mTOR signaling cascade. Conventionally, this molecular axis is heralded for its neuroprotective, anti‐apoptotic, and pro‐survival roles under physiological conditions and mild ischemic preconditioning [40, 41]. However, its function may flip under the extreme adrenergic and oxidative stress characteristic of hemorrhagic lesions [42]. Several mechanisms may account for this switch. First, PI3K/AKT activation via G protein‐coupled receptor βγ subunits can suppress apoptosis and maintain plasma membrane integrity, paradoxically enabling a “vital NETosis” program in which neutrophils release chromatin without immediate lysis. Second, persistent AKT activation may promote glycolytic reprogramming and mitochondrial ROS production, which in turn drive chromatin decondensation and granule protein release. Third, mTOR‐mediated inhibition of autophagy—normally a survival mechanism—can paradoxically accelerate NET release by impairing the clearance of damaged organelles and promoting the accumulation of NET‐inducing factors. Our in vitro data, reinforced by pharmacological activation utilizing SC79, demonstrate that artificially stimulating AKT forcefully reignites NET formation despite upstream adrenergic blockade. This metabolic hijacking is corroborated by recent immunological studies showing that PI3K/AKT/mTOR hyperactivation serves as a metabolic engine driving NET formation during severe systemic inflammation [43]. Foundational studies by Wu et al. and Xian et al. confirmed that PI3K/AKT transmits extracellular stress signals into the chromatin decondensation machinery [20, 40]. However, we note that our evidence is primarily pharmacological. Although these data support a contributory role for PI3K/AKT/mTOR in NET‐associated cell death, they do not establish necessity or sufficiency. Genetic loss‐of‐function experiments (e.g., AKT1/2 knockdown or mTOR conditional deletion in neutrophils) are required to definitively determine whether this pathway is essential. This distinction is important: PI3K/AKT/mTOR may function as a permissive amplifier rather than an obligatory driver of NETosis, and the therapeutic implication of targeting this axis should be interpreted with this caveat in mind.

Pharmacological silencing of the adrenergic storm presents a highly effective strategy for reversing structural and cognitive deficits. By utilizing the pan‐adrenergic receptor inhibitor cocktail PPA, we achieved a robust rescue of glymphatic function. This intervention successfully prevented the epinephrine‐induced PI3K/AKT/mTOR hyperactivation, halted NET generation, and repolarized AQP4 channels. Although contemporary therapeutic explorations have successfully focused on direct NET degradation using recombinant DNase I or PAD4 inhibitors to ameliorate neuroinflammation, our application of the PPA cocktail acts as an upstream protective shield. By cutting off the sympathetic overstimulation, we prevent the neuro‐immune‐vascular dysregulation at its origin. Furthermore, the profound behavioral recovery observed in spatial memory and motor function explicitly ties the restoration of macroscopic fluid homeostasis to the suppression of the systemic sympathetic surge.

Despite these findings, several limitations within the current experimental paradigm must be acknowledged. First, while rodent models effectively replicate acute neuroinflammatory responses, they may not perfectly encapsulate the exact volumetric proportionality of human CSF dynamics. Second, the systemic administration of pan‐adrenergic inhibitors requires careful clinical calibration to manage cardiovascular hemodynamics. Prazosin, propranolol, and atipamezole can substantially alter blood pressure, heart rate, cerebral perfusion, arterial pulsatility, and possibly CSF dynamics. Atipamezole may additionally alter arousal and catecholamine release. All of these variables can independently influence glymphatic tracer movement and behavioral performance. We must acknowledge that our current study did not systematically monitor these cardiovascular parameters. To address this critical gap in future work, we propose the following testable strategies upon future upgrades and iterations of animal experimental equipment: (1) continuous monitoring of mean arterial pressure, heart rate, and intracranial pressure during PPA administration using telemetry or intra‐arterial catheters; (2) assessment of cerebral perfusion and arterial pulsatility using laser Doppler flowmetry or high‐frequency ultrasound; (3) measurement of CSF production rate using ventriculocisternal perfusion to distinguish direct effects on CSF dynamics from secondary hemodynamic effects; and (4) intrathecal or cisterna magna delivery of PPA to minimize systemic exposure while achieving local adrenergic blockade. Third, only male Sprague–Dawley rats were used in this study. Sex differences in adrenergic signaling, immune cell trafficking, NET formation, and glymphatic function are increasingly recognized. Female‐derived neutrophils exhibit faster and more robust NETosis compared to male‐derived neutrophils, with up to 33% more DNA release. Estrogen can promote NET formation during infection while suppressing excessive NETs in males. Additionally, microglia in male rodents are already in a more activated state and are more responsive to neuroinflammatory stimuli. These sex‐based differences could significantly influence the generalizability of our findings. We justify the use of male rats by the need to eliminate hormonal cycle variability in this initial mechanistic study; however, we explicitly acknowledge that validation in both sexes is essential, and future studies should include ovariectomized and hormone‐replaced female rats to dissect the role of estrogen in adrenergic–NETs–AQP4 signaling. Fourth, our pharmacological approach utilized pan‐adrenergic receptor inhibitors rather than receptor‐selective antagonists or genetic knockdown. Receptor‐selective antagonists (e.g., the β_2_‐AR antagonist ICI‐118,551 or α₁‐AR antagonist prazosin alone) or neutrophil‐specific adrenergic receptor knockdown experiments would help establish which receptor subtype mediates the adrenergic effects on NET formation. Given that β_2_‐AR is the best‐established adrenergic receptor subtype on neutrophils, future studies should prioritize β_2_‐AR–selective blockade to determine whether the NET‐inducing effects of epinephrine are β_2_‐AR–dependent, while also testing α₁‐AR and α_2_‐AR contributions to rule out non‐specific effects. Finally, while scRNA‐seq reveals profound heterogeneity, the precise spatiotemporal interactions between specific neutrophil subclusters and various glial populations require further elucidation via high‐resolution spatial transcriptomics. Future investigations must therefore incorporate higher‐order primate models and highly specific targeted delivery systems to fully validate these neuro‐immune mechanisms.

## 5 Conclusion

This research report delineates a highly integrated pathological cascade dictating brain recovery following acute hemorrhagic injury. The acute systemic adrenergic storm drives a highly mobilized neutrophil subset to rapidly populate the brain. Associated with epinephrine‐induced activation of the intracellular PI3K/AKT/mTOR signaling axis, these neutrophils execute an overwhelming wave of NETs. The resulting rigid DNA‐histone webs barricade cerebrospinal fluid flow channels and destroy AQP4 polarization at the gliovascular interface. This dysregulation of the glymphatic system clinically results in massive secondary hydrocephalus and neurocognitive decline. Silencing this adrenergic storm via a pan‐adrenergic inhibitor cocktail successfully dampens the PI3K/AKT/mTOR metabolic engine, dismantles NETs, repolarizes AQP4, and restores convective glymphatic flow. Moving forward, precision neurocritical care aiming to intercept the post‐stroke adrenergic storm and its downstream NETs holds promise for the long‐term structural preservation of the brain's fluid clearance networks.

## Author Contributions

YJ.Y, X.W., and GL.X. were responsible for the conception design. YJ.Y, YJ.Z., QO.Y, H.N., and YX.Y. performed the experiments. YJ.Y and YJ.Z. wrote the initial manuscript. YJ.Y, JQ.W., KY.W., P.L., and YF.C. prepared the figures. YX.Y., ZK.L., J.H., and ZP.Z. analyzed and interpreted the data. GL.X., X.W., and ZJ.S. conducted a critical review of the paper. GL.X. and X.W. provided financial support. All authors read and approved the final manuscript.

## Funding

This work was supported by grants from National Natural Science Foundation of China (No. 82371362) and National Health Commission of China (No. GWJJZX20251001014).

## Ethics Statement

All practices on animals were performed in line with the National Institutes of Health Guide for the Care and Use of Laboratory Animals. The experiments were conducted in accordance with the approved guidelines and received approval from the Experimental Animal Welfare and Ethics Review Committee at Xiangya hospital of Central South University (202509140).

## Conflicts of Interest

The authors declare no conflicts interest.

## Supporting information


**Figure S1:** Experimental timeline.
**Figure S2:** scRNA‐seq data Analysis 1. (A) UMAP visualization of all integrated single cells, colored by groups. (B) UMAP specifically highlighting the spatial distribution of neutrophils compared to all other cell types across both groups. (C) Absolute total counts of neutrophils detected in the dataset. (D) Overall proportion of neutrophils relative to all sequenced cells. (E) Comparing the proportion of neutrophils within the Control group (0.72%) and the HS group (5.4%). (F) Distribution and relative composition of the different neutrophil sub‐clusters (0 through 4) separated by Control and HS groups. (G, H) Quantitative analyses of specific neutrophil subpopulations. Bar graphs (counts) and pie charts (percentages) comparing the abundance of Sub‐cluster 0 (G) and Sub‐Cluster 1 (H) between the Control and HS groups.
**Figure S3:** scRNA‐seq data Analysis 2. (A) Expression levels of representative core marker genes (*Osgin1, Clec4n, Grasp, Ahnak, Etv3*, and *Dock10*) across the 5 distinct neutrophil sub‐clusters (Identities 0–4). (B) UMAP feature plots illustrating the single‐cell expression distribution and spatial localization of the 6 representative marker genes within the neutrophil population. (C) Expression patterns of the core marker genes across the neutrophil sub‐clusters. (D) Expression patterns of the top 10 signature marker genes across all five neutrophil sub‐clusters (0 through 4). (E) Pseudo‐time trajectory analysis of the neutrophil sub‐clusters projected onto UMAP. (F) Global cell–cell communication network between neutrophils and other major cell types.
**Figure S4:** scRNA‐seq data Analysis 3. (A) Significant ligand‐receptor signaling interactions sent by neutrophils to target cell populations. (B–E) Inferred cell–cell communication networks where neutrophils serve as the signaling source, specifically detailing the: (B) Recruitment CCL Network, (C) Recruitment CXCL Network, (D) Endothelial damage TNF targeted network, and (E) Neuroinflammation SPP1 network.
**Figure S5:** MRI T2 sequence. (A) MRI T2 sequence of each group. (B) 3D rendering of each group.
**Figure S6:** Primary images for flow cytometry.
**Figure S7:** Original photo of ELISA. (A) Epinephrine. B: MPO‐DNA.
**Data S1:** Supplementary Methods.
**Table S1:** Numbers of animal used in experiment.
**Table S2:** Antibodies used in immunofluorescence staining.
**Table S3:** Antibodies used in western blot.

## Data Availability

In this study, the raw data are available from the corresponding author upon reasonable request and through collaborative investigations. All other data needed to evaluate the conclusions in the paper are presented in the paper or the Supporting Information. The scRNA‐seq raw data have been deposited in the NCBI Sequence Read Archive (SRA) under accession code PRJNA705094. The processed count matrices and associated metadata are available in the NCBI Gene Expression Omnibus (GEO) under accession code GSE167593.
