# Supplementary material for: Pan‐Adrenergic Inhibition Alleviates Post‐Hemorrhagic Hydrocephalus by Attenuating Neutrophil Extracellular Traps‐Mediated Glymphatic Dysfunction
Source: CNS Neurosci Ther. 2026 Nov 7;32(11):e71259. doi: 10.1002/cns.71259 (PMC13743993; doi:10.1002/cns.71259)
Supplement: Supplementary file 1 [file CNS-32-e71259-s001.docx]

**Supplementary Material**

**Supplementary Methods:**

**Intraventricular hemorrhage model:** Briefly, the rats were anesthetized with 10% pentobarbital (40mg/kg) and secured on a stereotaxic instrument (RWD, China). Autologous blood (200 μL) was injected in right lateral ventricle with a microsyringe and microinfusion pump at a speed of 20 μL/min (coordinates: bregma, posterior 0.72 mm; lateral 1.60 mm; depth 4.00 mm). The device was left in place for 5 minutes before we closed the cranial foramen and scalp incision[1]. Throughout the procedure, the rats were maintained on a heating pad to keep their body temperature constant, while the operator closely monitored their respiratory status. Noninvasive blood pressure and heart rate were measured at various postoperative time points using a noninvasive blood pressure monitor (BP-2010A, Softron, China).

**Drug injection:** Pan-adrenergic inhibitor cocktail contained Prazosin, Propranolol and Atipamezole (PPA)[2]. The aim was to broadly inhibit adrenergic receptors rather than to target catecholamine release. Prazosin (CM00858, Proteintech, China) is an inhibitor of α1 and dissolved in dimethyl sulfoxide (DMSO) and then diluted with saline (0.9% NaCl). The dosage and method of administering Prazosin were consistent with previous studies, with a dose of 5mg/kg injected through intraperitoneal once a day. Propranolol (CM16191, Proteintech, China) is an inhibitor of β and dissolved in DMSO and then diluted with saline (0.9% NaCl). The dosage and method of administering Propranolol were consistent with previous studies, with a dose of 5mg/kg injected through intraperitoneal once a day. Atipamezole (CM14700, Proteintech, China) is an inhibitor of α2 and dissolved in DMSO and then diluted with saline (0.9% NaCl). The dosage and method of administering Atipamezole were consistent with previous studies, with a dose of 0.5mg/kg injected through intraperitoneal once a day. PMA (Phorbol 12-myristate 13-acetate, HY-18739, MCE, United States) is an activator of NETs and dissolved in DMSO and then diluted with saline (0.9% NaCl). The dosage and method of administering PMA were consistent with previous studies, with a dose of 0.5mg/kg injected through intracisternal magna once a day. DNase Ⅰ (HY-108882, MCE, United States) is an inhibitor of NETs and dissolved in dimethyl sulfoxide (DMSO) and then diluted with saline (0.9% NaCl). The dosage and method of administering DNase Ⅰ were consistent with previous studies, with a dose of 6000U/kg injected through intraperitoneal once a day. They would be dosed postoperative and continue to third day. Rats in the sham and IVH groups received the same vehicle without drug as a control.

**single-cell RNA sequencing (scRNA-seq) data analysis**: Publicly available scRNA-seq data was obtained from the GEO database (GSE167593). Brains from healthy mice (n = 8), ischemic stroke mice (n = 8), and hemorrhagic stroke mice (n = 8) were used. Approximately 12,000 cells were loaded onto each 10X Genomics channel. Cells with UMI/gene numbers outside the limit of mean ± 2-fold standard deviation were filtered out. Cells with >30% mitochondrial gene counts were discarded. UMI/gene number outlier filtering was used to remove likely multiplet. Batch effects were removed using mutual nearest neighbors implemented in the R package batchelor. For scRNA-seq, differentially expressed genes were identified using p < 0.05 and log2 fold change > 0.58. Following preliminary quality control and filtering, the processed data was analyzed using the Seurat R package. Uniform Manifold Approximation and Projection (UMAP) was utilized for visualization of global cell types and sample distributions. Differences in the relative proportions of specific cell populations between groups were statistically evaluated using the Chi-square test. For high-resolution sub-clustering, the neutrophil population was computationally isolated. The subset underwent re-identification of highly variable features, scaling, Principal Component Analysis (PCA), and unsupervised clustering using FindNeighbors and FindClusters functions. Differentially expressed marker genes for each neutrophil sub-cluster were identified utilizing the FindAllMarkers function. Furthermore, to evaluate the biological function of neutrophils, a Neutrophil Extracellular Traps (NETs) potential score was calculated for each cell using the AddModuleScore function based on a curated signature gene set.

The signature gene set was defined as Padi4, Elane, Mpo, S100a8, S100a9, Camp, Ltf, Mmp9, Cybb, Ncf1, Ncf2, Rac2. Pseudotime trajectory inference of the neutrophil sub-clusters was performed using the slingshot R package to map their developmental or activation lineages. Finally, intercellular communication networks (e.g., CCL, CXCL, and TNF signaling pathways) between neutrophils and other cell types were inferred and visualized using the CellChat package[3, 4]. The scRNA-seq dataset used in this study was derived from a pre-merged Seurat object in which individual sample identifiers (orig.ident) were not retained. Consequently, biological replicate information was unavailable, and cell-level statistical tests would constitute pseudoreplication. Therefore, all scRNA-seq results are presented descriptively, and no donor-level statistical inference is claimed. Cell proportions and gene expression are

reported as observed values without P values.

**Brain section:** Rats were euthanized with an overdose of anesthesia, followed by perfused with 4% paraformaldehyde. After the perfusion, the brain was gently removed from the skull. They were placed in 4% paraformaldehyde and sliced after paraffin embedding (Leica, Germany).

**Immunofluorescence (IF) staining:** Brain sections were washed in phosphate buffer saline (PBS) and blocked with 3% bovine serum albumin, 0.2% Triton, and 0.05% Tween 20 in PBS for 1.5 h at room temperature. The primary and secondary antibodies were incubated overnight at 4 °C and for 2 h at room temperature, respectively. The primary antibodies used for IF were listed in **Table S2.**

**Transmission electron microscopy (TEM):** The brain tissues fixed in glutaraldehyde were rinsed with cold PBS, fixed in osmium tetroxide, dehydrated sequentially in gradient acetone. The tissue blocks, which had been adequately soaked overnight at 37℃, were placed in capsules filled with embedding agent and polymerized in a constant temperature (60℃) oven for 48 hours. Afterwards, the tissue was sliced with an ultrathin sectioning machine (thickness about 80-100 nm), and the satisfactory sections were retrieved and double-stained with 3% uranyl acetate as well as lead nitrate. Eventually observed them under TECNAI G 20 TWIN (FEI, America).

**Open field test (OFT):** Rats were put in an (50×50×50cm) and allowed to freely explore without any confinement for 10 minutes. The time they spent in inner zone and number of inner zone they crossing were recorded and analyzed by TopScan (Gene＆I, China).

**Novel object recognition (NOR):** Firstly, a rat was put in the center of open field with two identical objects placed equidistant from the sidewall and allowed to freely explore without any confinement for 10 minutes. Four hours later, one of the old objects would be replaced and tested again for 10 minutes. The time they spent on novel or old object was recorded and analyzed by TopScan (Gene＆I, China). The recognition index was calculated as: novel exploration time/total exploration time. The discrimination index was calculated as: (novel exploration time – old exploration time)/total exploration time.

**Morris water maze (MWM):** MWM consisted of 5 continuous days for training and 1 day for testing. For training on the first day, the rats were trained to seek a visible platform within 60s and stay on the platform for 30s. If the platform was not found within 60s, the rat was gently guided to reach the platform and remained there for 30s, and recorded as 60s. For training on the second to fifth days, the rats were trained to seek a hidden platform and others same as the first day. During the training phase, the rats were trained from the first to the fourth quadrant four times a day. The test was conducted on the sixth day, with the platform removed, and the rat was placed on the opposite side of the target quadrant (quadrant with platform). The latencies to the platform they spent, time they spent in target quadrant and number of platform they crossing were recorded and analyzed by TopScan (Gene＆I, China).

**Intracisternal magna injection and Inflow:** Rats were anesthetized with 10% pentobarbital (40mg/kg) and secured on a stereotaxic instrument. The rats were maintained in the prone position throughout the entire procedure. A heating pad was used to maintain body temperature, and the depth of anesthesia was closely monitored by the operator. The incision of the skin covering the occipital bone was operated to expose the dura mater of the cisterna magna. A polyethylene tube filled with a needle containing 2.5% Rhodamine B isothiocyanate-Dextran-average mol wt-70,000 (RITC-D 70K) (R9379, Sigma, America) was inserted parallelly to the skull, penetrating the dura mater to a depth of approximately 2 mm. RITC-D70K was then injected through a microinfusion pump at a speed of 5ul/min for 10 min. Rats were perfused with 4% paraformaldehyde 30 minutes after the injection. After the perfusion, the brain was removed and sectioned into four coronal brain sections at bregma +1.6 mm, -2.4mm, -3.2mm, and -4.0mm with a thickness of 100 μm using a cryostat microtome. RITC-D70K was selected because its molecular weight (70 kDa) is similar to that of albumin (66 kDa), it is restricted to the perivascular space, and its transport depends primarily on convection rather than diffusion, making it the gold standard for assessing glymphatic function[5].

**Intrahippocampal injection and Outflow:** Rats were anesthetized with 10% pentobarbital (40mg/kg) and secured on a stereotaxic instrument. The rats were maintained in the prone position throughout the entire procedure. A heating pad was used to maintain body temperature, and the depth of anesthesia was closely monitored by the operator. 2.5% RITC-D70K was injected into hippocampal with a microsyringe and microinfusion pump at a speed of 0.5 μL/min for 10 min (coordinates: bregma, posterior 3.20 mm; lateral 2.00 mm; depth 3.00 mm). Rats were perfused with 4% paraformaldehyde 60 minutes after the injection. After the perfusion, the brain was removed and sectioned into four coronal brain sections at bregma -1.6 mm, -2.4mm, -3.2mm, and -4.0mm with a thickness of 100 μm using a cryostat microtome[6].

**Cell culture:** Primary neutrophils were isolated from the bone marrow of SD rats using discontinuous density gradient centrifugation. Briefly, intact femurs and tibias were excised, and the marrow cavity was flushed with cold PBS. Following red blood cell (RBC) depletion using a standard lysis buffer, the resulting cell suspension was gently layered onto a three-layer Percoll density gradient (52%, 64%, and 72%) and centrifuged at 1000 × g for 30 minutes at 4°C without braking. The neutrophil-enriched fraction at the 64%/72% interface was carefully harvested, washed twice with cold PBS, and resuspended in RPMI-1640 medium. To construct an in vitro adrenergic storm model, an extensive literature search was conducted, in which each group of cells was treated with 10μM Epinephrine (HY-B0447B, MCE, United States) dissolved in PBS. The cells were divided into six groups in experiment 1: control, Epi (Epinephrine), Epi+PPA (5μM Prazosin, 5μM Propranolol and 0.5μM Atipamezole), Epi+Prazosin (5μM Prazosin), Epi+Propranolol (5μM Propranolol) and Epi+Atipamezole (0.5μM Atipamezole). The cells were divided into four groups: control, control+PPA, Epi, Epi+PPA, Epi+PPA+SC79 (AKT activator, 10μM) and Epi+DNase Ⅰ (NETs inhibitor, 1000U/mL). Rat astrocytes (KC9642, Kinlogix Biotech Co., Ltd., China) were cultured and passaged in complete medium for rat astrocytes (KC9642M, Kinlogix Biotech Co., Ltd., China). Mouse lymph node endothelial cells (CL-0221, Procell Biotech Co., Ltd., China) were cultured and passaged in complete medium for mouse lymph node endothelial cells (CM-0221, Procell Biotech Co., Ltd., China). After three sub cultures, the cells were used for subsequent experiments. Isolated neutrophils were plated at a density of 9×106 cells per well in a 6-well plate. Then, 500 nM PMA (CM00437, Proteintech, China) was added to each well and incubated in a 37 °C, 5% CO2 incubator for 3 h[7]. The samples were collected by scraping the cells and transferred to new centrifuge tubes, which were then centrifuged at 450 g and 4 °C for 10 min. The resulting supernatant contained NETs, whose concentration was measured using the Quant-iT Picogreen dsDNA Assay Kit (P9740, Solarbio, China). Purified NETs were co-cultured with astrocytes at 1000 ng/mL[8].

**CCK8:** Cell viability was assessed using the Cell Counting Kit-8 (CCK-8) assay. Briefly, cells were seeded into 96-well plates at a density of 5 × 10³ cells/well and incubated overnight. Following the indicated treatments, 10 μL of CCK-8 solution was added to each well and incubated for an additional 2 hours at 37°C in the dark. The absorbance was measured at 450 nm using a microplate reader.

**Apoptosis:** Apoptosis was assessed using the Annexin V-FITC/PI cell apoptosis detection kit (MA0220-2, meilunbio). Cells in the different experimental groups were trypsinized without EDTA, suspended in binding buffer, and incubated with Annexin V-FITC and PI for 15 min. Flow cytometry (LongCyteC2060, CHALLEN BIO, China) was then performed to analyze the results.

**Image analysis:** Stained sections were obtained by DS-Fi3 (Nikon Corporation, Japan), and images were captured with constant exposure time, offset, and gain for each staining marker. The area of positive signal was measured using ImageJ with grayscale threshold analysis. The minimum and maximum intensity settings for each staining marker remained constant. For the brain sections, the percentage area of positive signal for GFAP, Iba-1, RITC-D 70K, Cit-H3, MPO and NE was calculated by dividing the positive signal area by the total area of the image through ImageJ. All quantitative analyses were performed in a blinded fashion by two experimenters that was unaware of the sample identity.

**Reference:**

1.	Yang, Y., et al., *Therapeutic Hypothermia Alleviates Hydrocephalus and Neurological Dysfunction Post Intraventricular Hemorrhage by Enhancing Drainage of Glymphatic-Meningeal Lymphatic-Deep Cervical Lymphatic System.* CNS Neurosci Ther, 2026. **32**(1): p. e70740.

2.	Sun, Q., et al., *Enhancing glymphatic fluid transport by pan-adrenergic inhibition suppresses epileptogenesis in male mice.* Nat Commun, 2024. **15**(1): p. 9600.

3.	Shi, X., et al., *Stroke subtype-dependent synapse elimination by reactive gliosis in mice.* Nat Commun, 2021. **12**(1): p. 6943.

4.	Huang, H., et al., *Pro-repair macrophages driven by CGRP rescue white matter integrity following intracerebral hemorrhage.* J Neuroinflammation, 2025. **22**(1): p. 161.

5.	Dong, S., et al., *Cannabidiol Alleviates Neurological Deficits After Traumatic Brain Injury by Improving Intracranial Lymphatic Drainage.* J Neurotrauma, 2024. **41**(15-16): p. e2009-e2025.

6.	Liu, M., et al., *Exogenous interleukin 33 enhances the brain's lymphatic drainage and toxic protein clearance in acute traumatic brain injury mice.* Acta Neuropathol Commun, 2023. **11**(1): p. 61.

7.	Hoppenbrouwers, T., et al., *In vitro induction of NETosis: Comprehensive live imaging comparison and systematic review.* PLoS One, 2017. **12**(5): p. e0176472.

8.	Zhang, Q., et al., *Neutrophil extracellular trap-mediated impairment of meningeal lymphatic drainage exacerbates secondary hydrocephalus after intraventricular hemorrhage.* Theranostics, 2024. **14**(5): p. 1909-1938.

**Table S1: Numbers of animal used in experiment.**

**Part 1:**

| Tests | sham | sham+PPA | IVH | IVH+PPA | IVH+PPA+PMA | IVH+DNase Ⅰ |
| --- | --- | --- | --- | --- | --- | --- |
| MRI | 6 | 6 | 6 | 6 | 6 | 6 |
| IF | 6 | 6 | 6 | 6 | 6 | 6 |

Total number: **72**.

**Part 2:**

| Tests | sham | sham+PPA | IVH | IVH+PPA | IVH+PPA+PMA | IVH+DNase Ⅰ |
| --- | --- | --- | --- | --- | --- | --- |
| OFT  NOR  MWM | 6 | 6 | 6 | 6 | 6 | 6 |

Total number: **36**.

**Part 3:**

| Tests | sham | sham+PPA | IVH | IVH+PPA | IVH+PPA+PMA | IVH+DNase Ⅰ |
| --- | --- | --- | --- | --- | --- | --- |
| Inflow | 6 | 6 | 6 | 6 | 6 | 6 |
| Outflow | 6 | 6 | 6 | 6 | 6 | 6 |
| MRI | 6 | 6 | 6 | 6 | 6 | 6 |

Total number: **108**.

**Part 4:**

| Tests | sham | sham+PPA | IVH | IVH+PPA | IVH+PPA+PMA | IVH+DNase Ⅰ |
| --- | --- | --- | --- | --- | --- | --- |
| IF | 6 | 6 | 6 | 6 | 6 | 6 |
| TEM | 3 | 3 | 3 | 3 | 3 | 3 |

Total number: **54**.

**Part 5:**

| Tests | sham | IVH | IVH+PPA | IVH+PPA+PMA | IVH+DNase Ⅰ |
| --- | --- | --- | --- | --- | --- |
| IF | 6 | 6 | 6 | 6 | 6 |
| WB | 3 | 3 | 3 | 3 | 3 |

Total number: **45**.

**All rats used in experiment were 348 with 10.5% mortality, and 315 rats survived and sacrificed for experiment.**

**Table S2: Antibodies used in immunofluorescence staining.**

| **Antibody** | **Dilution** | **Source** | **ID** |
| --- | --- | --- | --- |
| rabbit anti-glial fibrillary acidic protein (GFAP) | 1:200 | Proteintech | 16825-1-AP |
| rabbit anti-ionized calcium binding adaptor molecule 1 (Iba-1) | 1:1000 | Abcam | ab178846 |
| rabbit anti-CD31 | 1:500 | Abcam | ab182981 |
| rabbit anti-aquaporin-4 (AQP4) | 1:200 | Proteintech | 16473-1-AP |
| rabbit anti-histone H3 (citrulline R2 + R8 + R17) (Cit-H3) | 1:200 | Abcam | ab281584 |
| rabbit anti-myeloperoxidase (MPO) | 1:500 | Proteintech | 22225-1-AP |
| rabbit anti-neutrophil elastase (NE) | 1:500 | Proteintech | 27642-1-AP |
| rabbit anti-lymphocyte antigen 6 complex (Ly6G) | 1:500 | Proteintech | 32346-1-AP |

**Table S3: Antibodies used in western blot.**

| **Antibody** | **Dilution** | **Source** | **ID** |
| --- | --- | --- | --- |
| rabbit anti-Cit-H3 | 1:1000 | Abcam | ab281584 |
| rabbit anti-MPO | 1:5000 | Proteintech | 22225-1-AP |
| rabbit anti-NE | 1:1000 | Proteintech | 27642-1-AP |
| rabbit anti-phospho phosphatidylinositol 3-kinase (pPI3K) | 1:2000 | Abmart | T40116 |
| rabbit anti-phosphatidylinositol 3-kinase (PI3K) | 1:2000 | Abmart | T40115 |
| rabbit anti-phospho AKT (pAKT) | 1:2000 | Abmart | T40067 |
| rabbit anti-AKT | 1:2000 | Abmart | T55561 |
| rabbit anti-phospho mammalian target of Rapamycin (pmTOR) | 1:1000 | Abmart | T56571 |
| rabbit anti-mammalian target of Rapamycin (mTOR) | 1:1000 | Abmart | T55306 |
| rabbit anti-GFAP | 1:2000 | Proteintech | 16825-1-AP |
| rabbit anti-Aquaporin-4 (AQP4) | 1:2000 | abcam | AB259318 |
| mouse anti-β-Dystroglycan (β-DG) | 1:2000 | SCB | sc-33702 |
| mouse anti-glyceraldehyde-3-phosphate dehydrogenase (GAPDH) | 1:500000 | Proteintech | 60004-1-Ig |
| rabbit anti-heat shock protein 90kDa (HSP90) | 1:10000 | Proteintech | 13171-1-AP |

**Figure S1:** **Experimental timeline.**

**Figure S2:** **scRNA-seq data analysis 1.**

**A**: UMAP visualization of all integrated single cells, colored by groups. **B**: UMAP specifically highlighting the spatial distribution of neutrophils compared to all other cell types across both groups. **C**: Absolute total counts of neutrophils detected in the dataset. **D**: Overall proportion of neutrophils relative to all sequenced cells. **E**: Comparing the proportion of neutrophils within the Control group (0.72%) and the HS group (5.4%). **F**: Distribution and relative composition of the different neutrophil sub-clusters (0 through 4) separated by Control and HS groups. **G**-**H**: Quantitative analyses of specific neutrophil subpopulations. Bar graphs (counts) and pie charts (percentages) comparing the abundance of Sub-cluster 0 **(G)** and Sub-cluster 1 **(H)** between the Control and HS groups.

**Figure S3: scRNA-seq data analysis 2.**

**A**: Expression levels of representative core marker genes (*Osgin1, Clec4n, Grasp, Ahnak, Etv3,* and *Dock10*) across the five distinct neutrophil sub-clusters (Identities 0–4). **B**: UMAP feature plots illustrating the single-cell expression distribution and spatial localization of the six representative marker genes within the neutrophil population. **C**: Expression patterns of the core marker genes across the neutrophil sub-clusters. **D**: Expression patterns of the top 10 signature marker genes across all five neutrophil sub-clusters (0 through 4). **E**: Pseudo-time trajectory analysis of the neutrophil sub-clusters projected onto UMAP. **F**: Global cell-cell communication network between neutrophils and other major cell types.

**Figure S4:** **scRNA-seq data analysis 3.**

**A**: Significant ligand-receptor signaling interactions sent by neutrophils to target cell populations. **B**-**E**: Inferred cell-cell communication networks where neutrophils serve as the signaling source, specifically detailing the: **(B)** Recruitment CCL Network, **(C)** Recruitment CXCL Network, **(D)** Endothelial Damage TNF Targeted Network, and **(E)** Neuroinflammation SPP1 Network.

**Figure S5. MRI T2 sequence.**

**A**: MRI T2 sequence of each group. **B**: 3D rendering of each group.

**Figure S6. Primary images for flow cytometry.**

**Figure S7. Original photo of ELISA.**

**A**: Epinephrine. **B**: MPO-DNA.
